# ModuleRole: A Tool for Modulization, Role Determination and Visualization in Protein-Protein Interaction Networks

**DOI:** 10.1371/journal.pone.0094608

**Published:** 2014-05-01

**Authors:** GuiPeng Li, Ming Li, YiWei Zhang, Dong Wang, Rong Li, Roger Guimerà, Juntao Tony Gao, Michael Q. Zhang

**Affiliations:** 1 MOE Key Laboratory of Bioinformatics; Bioinformatics Division and Center for Synthetic & Systems Biology, TNLIST, Department of Automation, Tsinghua University, Beijing, People’s Republic of China; 2 Stowers Institute for Medical Research, Kansas City, Missouri, United States of America; 3 Department of Molecular and Integrative Physiology, University of Kansas Medical Center, Kansas City, Kansas, United States of America; 4 Department of Molecular and Cell Biology, Center for Systems Biology, the University of Texas at Dallas, Richardson, Texas, United States of America; 5 Institucit of Molecular and Cell Biology, Center for System, Barcelona, Catalonia; 6 Departament d’Enginyeria Qu Cell Biology, Center for SystemBarce, Tarragona, Catalonia; 7 Bioinformatics Research Group, Key Laboratory of Intelligent Information Processing, Advanced Computer Research Center, Institute of Computing Technology, Chinese Academy of Sciences, Beijing, People’s Republic of China; 8 University of Chinese Academy of Sciences, Beijing, People’s Republic of China; 9 Department of Basic Medical Sciences, School of Medicine, Tsinghua University, Beijing, People’s Republic of China; Universita’ di Padova, Italy

## Abstract

Rapidly increasing amounts of (physical and genetic) protein-protein interaction (PPI) data are produced by various high-throughput techniques, and interpretation of these data remains a major challenge. In order to gain insight into the organization and structure of the resultant large complex networks formed by interacting molecules, using simulated annealing, a method based on the node connectivity, we developed ModuleRole, a user-friendly web server tool which finds modules in PPI network and defines the roles for every node, and produces files for visualization in Cytoscape and Pajek. For given proteins, it analyzes the PPI network from BioGRID database, finds and visualizes the modules these proteins form, and then defines the role every node plays in this network, based on two topological parameters Participation Coefficient and Z-score. This is the first program which provides interactive and very friendly interface for biologists to find and visualize modules and roles of proteins in PPI network. It can be tested online at the website http://www.bioinfo.org/modulerole/index.php, which is free and open to all users and there is no login requirement, with demo data provided by “User Guide” in the menu Help. Non-server application of this program is considered for high-throughput data with more than 200 nodes or user’s own interaction datasets. Users are able to bookmark the web link to the result page and access at a later time. As an interactive and highly customizable application, ModuleRole requires no expert knowledge in graph theory on the user side and can be used in both Linux and Windows system, thus a very useful tool for biologist to analyze and visualize PPI networks from databases such as BioGRID.

**Availability:**

ModuleRole is implemented in Java and C, and is freely available at http://www.bioinfo.org/modulerole/index.php. Supplementary information (user guide, demo data) is also available at this website. API for ModuleRole used for this program can be obtained upon request.

## Introduction

In recent years, high-throughput techniques have produced large networks of interacting molecules, which are represented as nodes linked by edges in complex graphs. In this context, the characterization of biological networks by means of graph topological properties has become very popular for gaining insight into the global network structure [1]. However, general software libraries for graph analysis such as JUNG [2], yFiles (http://www.yworks. com/) and NetworkX (https://networkx.lanl.gov/) etc. are not easily applied by the biological users. Specialized tools for the analysis of biological networks such as CentiBiN [3], tYNA/TopNet [4], and VisANT [5] etc. can calculate a set of topological parameters, but they do not offer valid way to gain insight into the structure, especially modules, of biological networks. The program CFinder [6], FastCommunity [7], MCL [8], SPICi [9], and Cohtop [10], and the popular open-source software Cytoscape [11] which offers almost dozen of plugins, including MCODE [12], NeMo [13], MINE [14], APCluster [8] and clusterMaker [15], can mine biological networks for clusters or modules [11], but none of them identifies the importance of nodes and assigns every node a role in a given network while doing clustering and modulization.

Here we developed ModuleRole, a user-friendly Java program which can modulize the PPI network using simulated annealing, a stochastic optimization technique that enables one to find ‘low cost’ configurations without getting trapped in ‘high cost’ local minima by introducing a computational temperature T and overcoming small cost barriers [16]. ModuleRole defines the role for every node based on two parameters Z-score and Participation Coefficient [16], and visualizes the modulized and role-determined network using popular molecular interaction network platform Cytoscape [11] and Pajek [17].

ModuleRole requires no expert knowledge in graph theory on the user side and the initial release of it was made available in July 2013. ModuleRole can analyze physical or genetic PPI network, or physical plus genetic network as a whole. ModuleRole can run these analysis on any individual sets of interactions given by users as well as on all proteins of each of more than 40 species in the different version of the PPI data in BioGRID database. To test the applicability of program ModuleRole to complex biological networks, we consider three applications in three different species: to find functional modules in cell polarity PPI network in budding yeast, to identify proteins key to metastatic prostate cancer in human being, and to verify functional modules in mouse PPI network. For users’ own interaction datasets, only the offline version of ModuleRole can be used (for example, cell polarity PPI network in budding yeast in the application of section 3.1).

### Motivation

So far a tool which can identify the importance of nodes in a given PPI network while doing modulization is still missing. ModuleRole is a tool to find modules in a given protein-protein interaction (PPI) network, to define the role of every protein and to visualize these modules and roles from any given PPI data source, such as BioGRID database, and the differentially expressed gene list from microarray and RNA-seq analysis. As an interactive and customizable application that requires no expert knowledge in graph theory from users, ModuleRole can be applied to any species in BioGRID database, and potentially, other databases containing molecule interactions.

## Results and Discussion

### Input

Although the data source for ModuleRole can be any given PPI data, the default one is BioGRID database, a general repository for physical and genetic interaction datasets from many different species [24]. BioGRID interaction data are 100% freely available to both commercial and academic users for research purposes. Though no warranty, it provides an important source to obtain the most up to date versions of physical and genetic interaction data.

**Figure 1:**
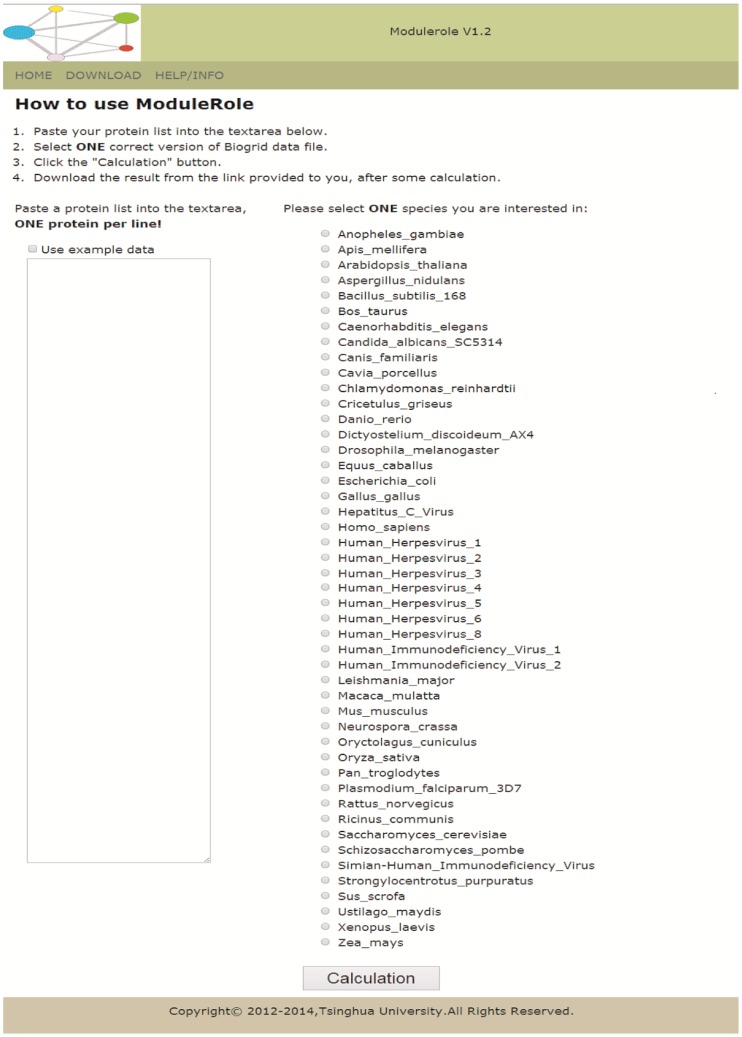
ModuleRole (online version) provides a user-friendly interface for users to find and to visualize modules, and to assign a role to each node, using BioGrid as default PPI database. The offline version can be used for analyzing larger datasets with thousands of proteins. ModuleRole can be used in both Linux and Windows operating system. The frame on the left is for user to input the list of proteins, and the list on the right side is all the species available so far for users to analyze the PPI network.

The ModuleRole program requires: (1) a list of protein names which is defined by user and (2) a tab file downloaded directly from BioGRID which contains PPI data. Multiple versions (from ver2.0.17 to current version) of BioGRID data for more than 40 species can be used as input for ModuleRole. Therefore, the interface of ModuleRole (Figure 1) is designed based on the requirement above. The input of ModuleRole is: (1) a list of proteins provided by user; (2) the species selected by user. After the data input by user, ModuleRole will analyze (1) physical PPI network, (2) genetic PPI network, (3) physical plus genetic network as a whole.

### Output

The output has been designed to detect and to visualize the details of interactions in every module. The output of ModuleRole can be divided into five parts: (1) modules in PPI physical and genetic network, and (2) role determination for every node (the description of the identified roles and their potential biological meaning can be found in Table 1); (3) files used for visualization in Cytoscape and Pajek; (4) eight report text files to show the details of modulization and role determination, including Participation Coefficient and Z-score computation; (5) other files, which will be used for developers, are not important for users.

**Table 1:** The description of the identified roles and their potential biological meaning.

Role	Node type	Hub ornon-hub	How is this node connected	Potential biological meaning
1	ultra-peripheralnodes	Non-hub	nodes with all their links withintheir module	Redundant with other proteins,or has a paralog. When deleted,the cell or species can survive welleven without any phenotypechanges.
2	peripheral nodes		nodes with most links withintheir module	
3	non-hub connectornodes		nodes with many links to othermodules	Protein interacts with proteins intwo or several different pathways.
4	non-hubkinless nodes		nodes with links homogeneouslydistributed among all modules	Protein involved in many pathwaysbut does not play key role.This kind of node seldom appears.
5	provincialhubs	Hub	hub nodes with vast majorityof links within their module	Essential protein plays key rolein one specific pathway.
6	connectorhubs		hubs with many links to mostof the other modules	Essential protein plays key rolein many pathways.
7	kinlesshubs		hubs with links homogeneouslydistributed among all modules	Seldom appears. Protein involvedin many pathways and play keyrole in several pathways.This kind of nodeseldom appears.

For more details about Input and Output, please check the supporting information and user guide.

### Visualization

ModuleRole offers two ways to visualize the results of modules and roles in a given interaction network: one is Cytoscape and the other is Pajek.

As a freely distributed software under the open-source GNU Lesser General Public License, Cytoscape and its plugins provide a powerful tool kit designed to help researchers to analyze and visualize multiple types of biological networks, in order to answer specific biological questions. ModuleRole produces two xgmml files for the visualization in Cytoscape: one file can visualize all proteins and their roles in every module while the other, coarse graining, focuses more on the connections among modules when overlooks the interaction details inside each module. Cytoscape allows users to set attribute values of nodes and edges, such as shape, color, size of nodes, and width of edges.

Pajek is a freely available program, for both Windows and Linux, to analyze and visualize large networks (http://vlado.fmf.uni-lj.si/pub/networks/pajek/). ModuleRole produces two NET files for visualization in Pajek: one is to show all proteins in each module while the other, coarse graining, focuses more on the connections among modules when overlooks the interaction details inside of every module. The parameters of visualized network, such as fonts and the colors of the nodes and interactions, can be configured in Pajek. Additionally, the title of the chart diagram, the labels of the axes, and the colors of the scatter points and gridlines can be configured. Once calculated and displayed, the network statistics can be saved into and reloaded from a text file in order to avoid recalculation. After visualization, Pajek will export the visualized network as chart images in the formats JPG/PNG/SVG or as tables in plain text files.

### Online and Off-line Version

The Online version can be tested at the website http://www.bioinfo.org/modulerole/index.php, which is free and open to all users without login requirement, with demo data provided by “User Guide” in the menu Help. Users are able either to download the zipped result to a local disk, or to bookmark the web link to the result page and access at a later time.

The Non-server version of ModuleRole, which is considered for high-throughput data with more than 200 nodes or user’s own interaction datasets, can be downloaded from the same website and run in Windows or Linux Operating system, either 32-bit or 64-bit.

### Application 1. Cell Polarity Network in Budding Yeast

Cell polarity has fundamental role in cell biology. Budding yeast *Sacchoramyces cerevisiae* is always used to study Cell polarity [25] [26]. The polar cortical domain (PCD) in budding yeast *Sacchoramyces cerevisiae* is a dynamic assembly of loosely interacting components which are composed of more than 100 different kinds of proteins.

**Figure 2:**
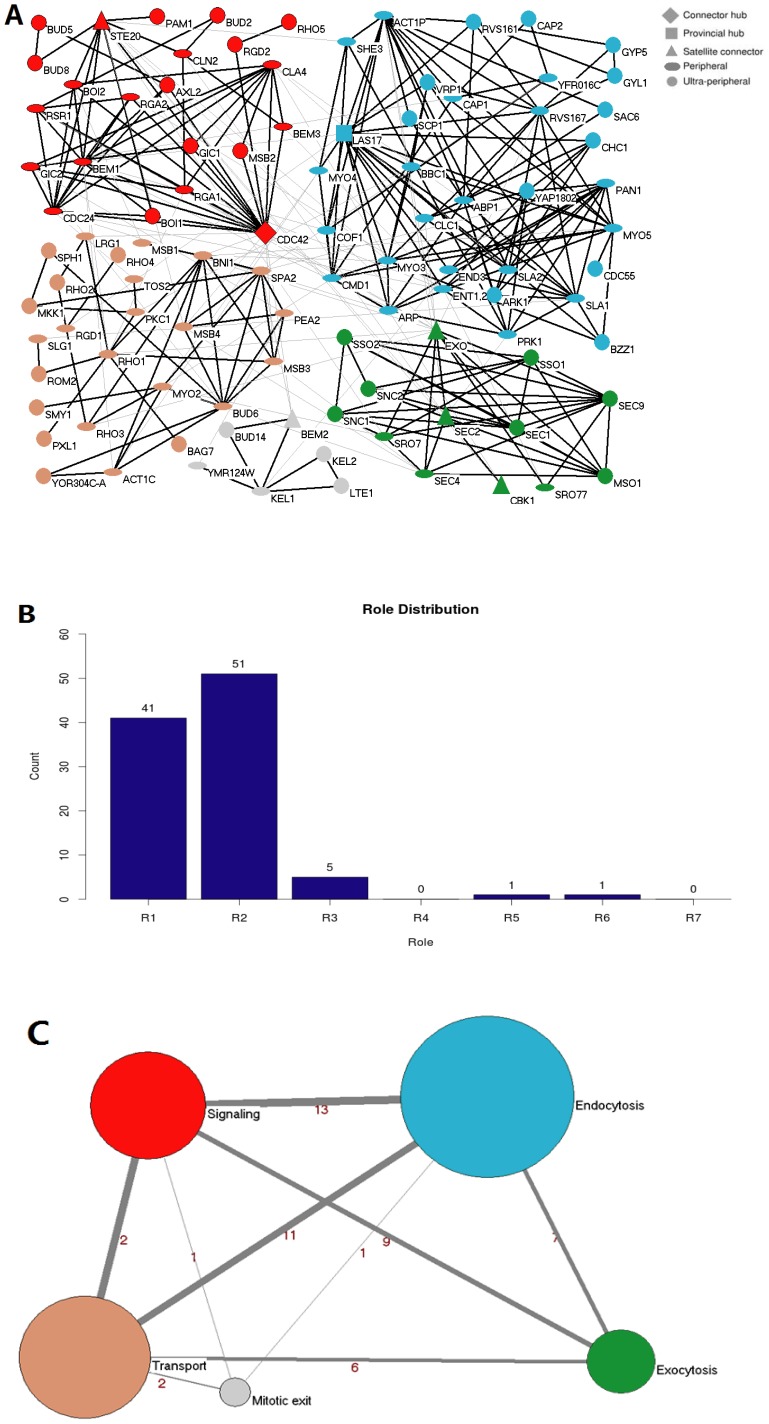
A network of polarity protein physical interactions. Polarity protein network contained 99 nodes and 302 linkages obtained from BioGrid database (version 2.0.51), visualized in Cytoscape (version 2.6.3). **A.** Visualization of the modularized PCD protein interaction network. Five modules with distinct functions constitute this network: Signaling (red), Transport (brown), Endocytosis (blue), Exocytosis (green), and Mitosis Exit regulation (light gray). Every node is a protein and the different node shape represents different universal role as indicated. The shape of every node indicates the role defined by ModuleRole, and the interpretation and potential biological meaning of these roles can be found in Table 1. The line between every two nodes represents the PPI interaction between two proteins. The bold lines indicate the PPI interactions inside a module, while the light gray lines indicate PPI interactions between modules. **B.** The role distribution of all 99 proteins shown in panel A. X axis is the role from 1 to 7, and Y axis shows the number of proteins which have the corresponding role. **C.** A coarse-graining of the network shown in panel A in which each module is represented by a single node, with edges representing interaction numbers between modules. The radius of the node is proportional to the node number in this module, and the thickness of the edges is proportional to the number of interaction among modules. The number written around the line indicates the interaction number between every two modules. The color of modules corresponds to the module color in panel A. Clearly this coarse-graining graph reveals much at global level, which is not easily seen in the original network in panel A.

Protein list of 111 proteins which physically localize in PCD area (Table S1) was selected manually and loaded into ModuleRole, with BioGRID database version 2.0.51 as input. As some proteins form a complex which should be treated as single node in the PPI network, such that ARP2 and ARP3 forms Arp2/3 complex to regulate actin polymerization [27], we changed slightly the list of polarity proteins (see the Note at the end of Table S1). Correspondingly, the BioGRID database version 2.0.51 was changed slightly as well. Here only the offline version of ModuleRole can be used because of the name changes of these several protein complexes (Table S2). With these two files as input, we got 302 physical interactions (Table S3) among 99 proteins, while other 12 of 111 proteins have no interactions inside of this polarity PPI network (Figure 2 A). Five consensus modules with specific function in the polarity protein PPI network in PCD were successfully unraveled (Figure 2 A) [28], and the corresponding role distribution (Figure 2 B) and coarse-graining graph (Figure 2C) are shown.

Proteins classified in the same module typically have known functions within a common sub-process related to cell polarity (Figure 2A). Four of the five modules correlate with functions known to be required for polarity and morphogenesis in yeast and are designated as Signaling, Transport, Endocytosis, and Exocytosis modules (Figure 2A). For example, in Transport module, module component BNI1, SPA2, PEA2, Bud6, SPH1, MSB3, and MSB4 forms polarisome which is required for retrograde transport of protein aggregates [29]. The Rho-type small GTPase CDC42 is activated by its guanine-nucleotide exchange factor CDC24 to polarize the cell for budding and mating. BEM1 interacts with CDC42, CDC24 and the effectors of CDC42, including the p21-activated kinase STE20 which functions in several signal transduction pathways, to function as a scaffold for cell polarity establishment - All of these proteins, which are important to initiate and establish cell polarity, belong to Signaling module. Similarly, many proteins in Endocytosis module are involved in endocytosis. The fifth module includes protein KEL2, which interacts with module member LTE1 and forms a complex with module component KEL1 to negatively regulate mitotic exit [30], thus correlates with function mitosis and is called Mitotic Exit module. Therefore, the modules defined by this program fit their biological function well.

For the roles assigned by ModuleRole, proteins indeed have corresponding importance of their functions in cell polarity establishment and maintenance. For example, CDC42, assigned by ModuleRole as connector hub (role = 6, that is, hub which plays key and fundamental role in the investigated PPI network, has many links to most of other modules ) with the highest degree (degree = 21) among all polarity proteins, is indeed the master regulator and essential small rho-like GTPase which controls the establishment and maintenance of cell polarity [31]; while CDC42 GTPase-associated protein Gic1, assigned an ultra-peripheral role (role = 1, with all its links within its module and no between-module links) by ModuleRole, is indeed not essential for its subcellular localization [32], and together with other proteins, forms one pathway, which is parallel to another and redundant, to link CDC42 to the actin cytoskeleton [33]. Taken together, these examples indicate that proteins with higher roles (role > = 3) can be essential, while protein with lower role (role = 1 or 2) can be non-essential and/or its function can be redundant, thus provide hint and direction to further investigate protein function and the mechanism of the related biological process.

Coarse-graining graph represents an important step towards extracting scale-specific information from complex networks. A coarse-graining graph of the polarity protein PPI network in PCD (Figure 2, panel B) helps people understand more about the interactions/relationship AMONG modules, which cannot be easily visualized in the original network shown in panel A of Figure 2. Coarse-graining graph overlooks the interaction details inside of every module, thus considerably simplifies the representation of the network as the nodes in its network do not need to be identified separately. In this graph (Figure 2, panel C), one can easily see that there are 13 interactions in total between Signaling module and Endocytosis module, 9 between Signaling and Exocytosis module, indicating that the communications among 3 modules (Signaling, Transport and Exocytosis) contribute more for polarity establishment and maintenance.

The example above shows that in budding yeast polarity protein PPI network, the modules and roles defined by this program fits their biological function well, and ModuleRole helps to establish a general connection between the topological concept of module/role in PPI network and protein’s biological functionality, thus offers a convenient way for users to predict the function and importance of unknown proteins in given PPI network, and to understand the mechanism of cell polarity establishment and maintenance [28]. Here we need to use this application as one example to point out that for users’ own interaction datasets, only the offline version of ModuleRole can be used.

### Application 2: To Find Key Genes Important for Metastatic Prostate Cancer

Prostate cancer is the second leading cause of cancer-related deaths in the United States among males. Prostate cancer metastasis occurs when cancer cells break away from the tumor in prostate and travel through the lymphatic system or bloodstream to other areas of the body, mostly lymph nodes and the bones [34]. Most prostate cancer–related deaths are due to advanced disease but not tumor in the prostate, thus it is very important to identify the key proteins during Prostate cancer metastasis [34].

Two data sets are analyzed in this application using R (Table S4) and Bioconductor [35]: GSE6919 [36] [37] and GSE32269 (http://www.ncbi.nlm.nih.gov/geo/) from GEO (the Gene Expression Omnibus) database [38] (Table S5). For the pipeline to analyze these two data sets, see Figure S1.

**Figure 3:**
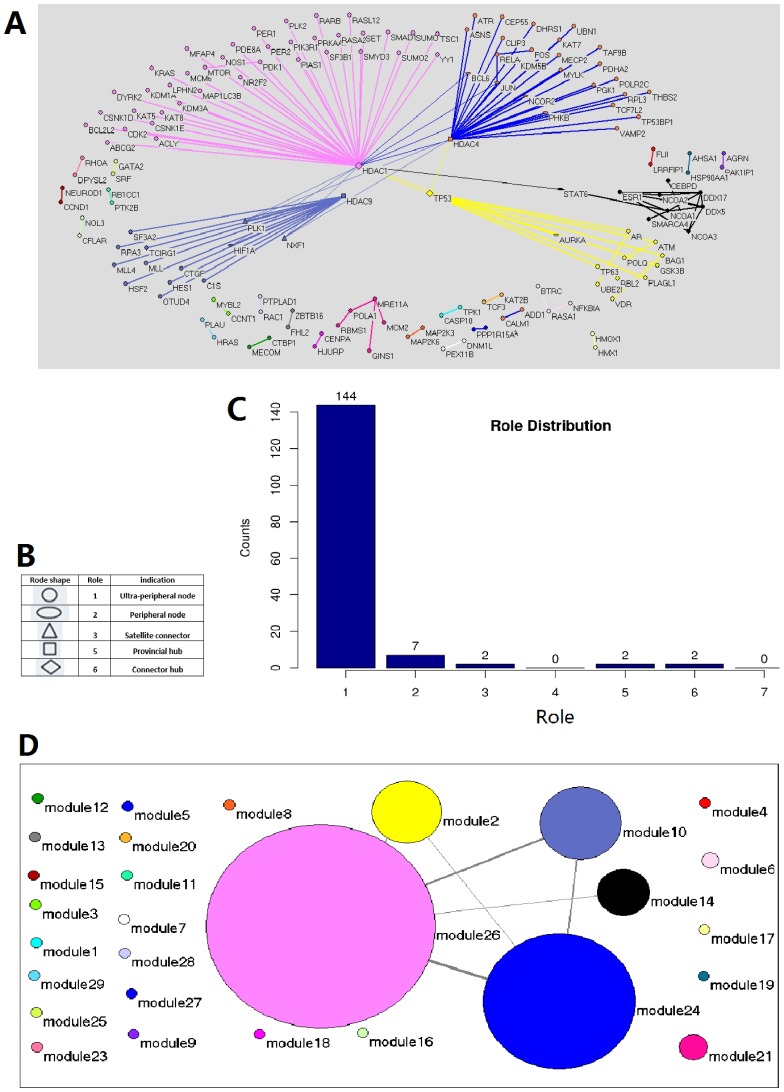
The identification of key genes important for prostate cancer metastatis. **A.** The modulization and role determination in the genetic interaction network of the 3998 differentially expressed genes between Metastatic state and Normal state in prostate cancer cells. Every node is a protein and the different node shapes represent different universal roles as indicated in panel **B.** The Line between every two nodes represents the PPI interaction between two proteins. The thicker lines indicate the PPI interactions inside a module, while the thinner ones indicate the interactions between modules. **C.** The role distribution of all proteins shown in panel **A.** X axis is the role from 1 to 7, and Y axis shows the number of proteins which have the corresponding role. **D.** A coarse graining of the network shown in panel A in which each module is represented by a single node, with edges representing interaction numbers between modules. The radius of the node is proportional to the node number in this module, and the thickness of the edges is proportional to the number of interaction among modules. The colors in panel D correspond to that in panel A. Both panel A and D were visualized in Pajek.

At first we classified these microarray samples into two different types: Metastatic prostate cancer (M), and Normal tissue (N). Next, in order to identify the differentially expressed genes, we compared the expression value in Metastatic prostate cancer and Normal tissue (M–N), respectively. The 3998 differentially expressed genes are shown in Table S6. Thereafter we reconstructed the PPI network from these 3998 genes, used ModuleRole to find the modules in PPI network and defined the genes key to Prostate cancer development and metastatic process (Figure 3, and Table 2).

**Table 2:** The genes which play key roles in Metastatic prostate cancer.

Gene name	Role
CREBBP HDAC1 HDAC3	6 (Connector hub)
EP300 MYC	5 (Provincial hub)
HIF1A JUN	3 (Satellite connector)
BRCA1 CDK2 KRAS MDM2 NCOA1NCOA3 SMAD3 TP53	2 (peripheral node)

In this list, CREBBP, a ubiquitously expressed gene defined as a connector hub (role = 6), a hub with many links to most of other modules, indicating that CREBBP plays key role in many pathways. This is consistent with the fact that CREBBP plays critical roles in many processes such as embryonic development, growth control, chromatin remodeling and other cell process [39]. JUN is assigned as non-hub connector (role = 3), which has many links to other modules; indeed, this protein is involved in many processes, such as the regulation by diverse extracellular stimuli, the progression through the G1 phase of the cell cycle, protection from apoptosis, and the early stage of tumor development, etc [40]. SMAD3, with role 2 indicating that such kind of proteins is redundant with other proteins, or has a paralog (Table 2), is indeed one of several human homologues of a gene [41].

These results are consistent with the map of central cancer pathways emphasized on prostate cancer and created through manual review of literature (http://cbio.mskcc.org/cancergenomics/prostate/pathways/), indicating that the roles defined by ModuleRole are meaningful and provide important hints to the mechanism of metastatic prostate cancer.

Besides roles, the modules defined by ModuleRole also have specific function or in the same pathway. For example, in one of these modules, module members HIF-1α and NF-κB mediate the down-regulation of the expression of another module member, PLK1 [42], with which module component PLK4 has the potential to interact and cross-activate in cells [43].

### Application 3: To Apply ModuleRole into PPI Network in Other Species such as Mouse

**Figure 4:**
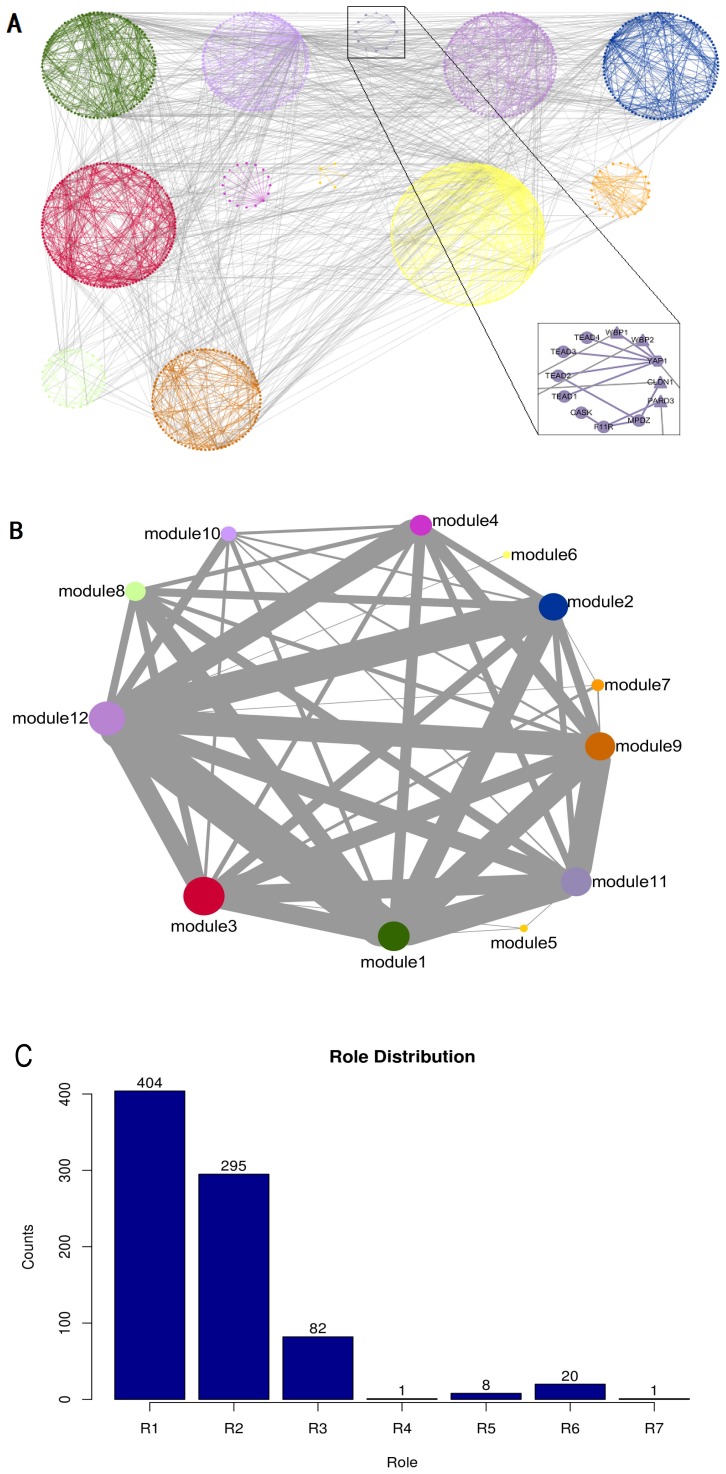
Analysis and visualization of a mouse physical PPI network with 813 nodes and 2222 interactions from BioGrid 3.2.103 (2 of these 813 proteins have no interactions inside of these proteins). **A.** The PPI network is defined by 12 different modules by ModuleRole, each protein is assigned a different role, and visualized in Cytoscape 3.0.2. Every node is a protein and the different node shape represents different universal role as indicated in Figure 2 and 3. The interpretation and potential biological meaning of role 1 to role 7 can be found in Table 1. The inset is to zoom into one of the modules which represents the Hippo signaling pathway regulated by cell polarity and cell junction proteins. **B.** A coarse-graining of the network in which each module is represented by a single node, with edges representing interaction numbers between modules. The radius of the node is proportional to the node number in this module, and the thickness of the edges is proportional to the number of interaction among modules. **C.** The role distribution of all 813 proteins in mouse physical PPI network.

ModuleRole can be applied to the PPI network of more than 40 species (Figure 1). As a further application, the modules and roles in mouse PPI network were defined by ModuleRole and visualized in Cytoscape 3.0.2 (Figure 4, panel **A**). 813 proteins (for the protein list, see Table S7) were the input of ModuleRole, and these proteins with 2222 PPI interactions were divided into 12 different modules with 7 defined roles.

The identified modules are related to their function closely. For example, most components in module 11 are closely related to the highly conserved Hippo signaling pathway which is regulated by cell polarity and cell junction proteins [44]: the module member YAP1, TREAD1-4 are the core components of Hippo pathway; module component YAP2 binds to adaptors WBP1 and WBP2 which are also members in this module, and YAP-WBP interaction plays a key role in the Hippo tumor suppressor pathway [45]. Cell junction protein MPDZ, a component in Module 11, was identified as interacting partners of core Hippo pathway components [46]. Claudins (CLDN1) constitute the major transmembrane proteins of tight junctions (TJs), while as a cell adhesion molecule, F11R is related to TJs. PARD proteins, including PARD3, are essential for asymmetric cell division and polarized growth. Module 11 is composed of all proteins mentioned above, indicating that the modules identified by ModuleRole indeed have specific functions, in the case of module 11, Hippo signaling pathway.

The coarse-graining graph of the mouse PPI network overlooks the interaction details in every module (Figure 4, panel B), showing that physical interactions are mainly between module 3, 6, 9, 12, and also between module 1 and 2.

Bone morphogenetic protein receptor type 2 (BMPR2) is essential for post-implantation physiology and fertility [47] thus has higher role (role 6, connector hub, with many links to most of the other modules). UbC is regarded a kinless hub (role 7) by ModuleRole, with links homogeneously distributed among all modules. Indeed, UbC(role 7) is thought to supplement the constitutive UbA genes in maintaining cellular ubiquitin (Ub) levels [48]. SMAD4, a major mediator of BMP and TGF-beta signaling, is required early in cerebellar development for maintaining the rhombic lip (RL) and generating subsets of RL-derived glutamatergic neurons [49], is also assigned higher role (role 6), while apoptosis regulator BCL2L2 is essential for spermatogenesis but appears otherwise redundant [50], thus has much lower role (role 1, ultra-peripheral node with all its links within its module).

The role distribution of all proteins in mouse physical PPI network (Figure 4, panel C) shows that most proteins have lower roles, i.e., around 50% of 811 proteins have role 1, 36.4% have role 2, while only 10.1% have role 3, 1% role 5, and 2.5% role 6, indicating that most nodes (86.4%) have all or most links within their module.

In addition to visualization, Cytoscape and its plugins open a new world for the topological analysis of biological networks. One example is plugin NetworkAnalyzer, which is used for the standard and advanced analysis of network topologies [51]. With the xgmml file produced by ModuleRole and loaded into Cytoscape, NetworkAnalyzer can further compute a number of topological parameters, such as node degree, clustering and topological coefficient, characteristic path length, betweenness centrality, etc (Figure S2), leading us to the better understanding of the structure of PPI network of mouse.

## Methods

### Modulization

A functional module is a discrete entity whose function is separable from those of other modules [18]. The goal of a module identification algorithm is to find the partition with largest modularity. Simulated annealing [19] is a stochastic optimization method that finds ‘low cost’ configurations by direct maximization of modularity M without getting trapped in ‘high cost’ local minima, which can be achieved by introducing a computational temperature T. When T is high, the method can explore configurations of high cost while at low T the system only explores low cost regions. Starting at high T and slowly decreasing it, the system descends gradually toward deep minima, overcoming small cost barriers [9] [20].

Given a network, for a certain partition P of the nodes into modules, the modularity M (P).

is defined as [21]:
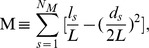



Where N_M_ is the number of modules, L is the number of links in the network, l_s_ is the number of links between nodes in module s, and d_s_ is the sum of the connectivity (degrees) of the nodes in module s. Modules (and the optimal number of modules) are typically identified by selecting the partition P* that maximizes M(P) [20] [22].

Two issues, however, make direct maximization of the modularity difficult: First, PPI network databases and their updated versions often contain numerous false positives and false negatives [23]. Second, two different partitions of the same network can have very similar values of modularity, so that by only looking at the partition with the largest modularity some potentially relevant information is lost [16]. A scheme [22] that combined network reconstructions with modularity maximization to control for error sensitivity in module identification was designed in ModuleRole to overcome these two issues.

The algorithm used in ModuleRole significantly outperforms the most algorithms [20], and ModuleRole can reliably identify modules whose nodes have as many as 50% of their connections outside of their own module [20]. In this program, one does not have to specify a priori the number of modules; instead, the number of modules is an outcome of the algorithm. The modularity value is given in the result file “report00_Summary.txt” (See Supporting Information for more details).

### Role Determination

While the majority of the topological parameters to measure interaction networks included in Cytoscape plugins and other programs are frequently used, ModuleRole additionally and efficiently computes two novel network properties (Z-score and Participation Coefficient) and defines the role every node plays in this network based on these two topological parameters. In particular, we have combined these two parameters with simulated annealing algorithm, to define both modules and roles in protein interaction network.

The idea to determine role is that nodes with the same role should have similar relative within-module connectivity. ModuleRole classifies nodes into universal roles according to their pattern of intra- and inter-module connections, based on two parameters: Participation coefficient and within module degree z-score [16] [20].

#### Participation coefficient

Participation coefficient *P_i_* of node *i* is defined as
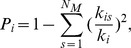



Where *k_is_* is the number of links of node *i* to nodes in module *s*, and *k_i_* is the total degree of node *i*. For node *i* in module *m,* participation coefficient measures the distribution of connections among the other modules. Nodes with all connections within their own module have a participation coefficient equal to zero whereas nodes with more connections to several other modules than to its own module have a participation coefficient closer to 1.

#### Within module degree z-score

For node *i* in module *m*, within module degree z-score measures how ‘well connected’ node *i* is to other nodes in the module, i.e., how different the number of connections to other nodes in the same modules with respect to the distribution of within module degrees for all of the nodes in the module. The within-module degree *z*-score is defined as:
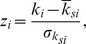



Where *κ_i_* is the number of links of node *i* to other nodes in its module *s_i_*, 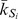 is the average of *κ* over all the nodes in *s_i_*, and 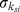 is the standard deviation of *κ* in *s_i_*.

These two parameters, showing how one node is positioned in its own module and with respect to other modules, define a z–P parameter space which has different areas to classify nodes into different roles [16] [20]. These two properties can be easily computed once the modules of a network are known. The description of the identified roles and their potential biological meaning can be found in Table 1.

## Conclusions

For a list of proteins defined by user, the program ModuleRole extracts both physical and genetic PPI network information from BioGRID database, finds the modules inside, defines the role of every protein based on two topological parameters Participation Coefficient and Z-Score, and visualizes these modules and roles in Cytoscape and Pajek. As a versatile and user-friendly tool to analyze BioGRID networks, this program adds node attributes (roles) and incorporates useful visualization settings to display and export the resulting modulization and roles. This is the first program which provides interactive and very friendly interface for biologists to find and visualize both modules and roles of proteins in PPI network. With all of these application together, we can find that ModuleRole can provide us new view of biological networks we are interested in, thus help us answer specific biological questions, with the help of third-party program such as Cytoscape and Pajek.

Although BioGRID interaction data, 100% freely available to both commercial and academic users for research purposes, offer a good source to support biological studies, it is interesting to explore the structure of interaction network in many other databases. Thus a new version of ModuleRole should be able to analyze the interaction network between protein and RNA (such as small RNA, lncRNAs (long noncoding RNAs) etc.) [52], and even the chromatin-chromatin interaction network produced by ChIA-PET [53], [54] and 3C-series data[55]–[58].

## Supporting Information

Figure S1 **The pipline for the analysis of metastatic prostate cancer data.** **A.** The workflow to find key genes for metastatic prostate cancer. **B.** The workflow to identify differentially expressed genes in data set GSE6919 and GSE32269.
(TIF)

Figure S2 **The plugin NetworkAnalyzer was used as an example to further analyze the xgmml file loded into Cytoscape.**
(TIF)

Table S1 **List of 111 polarity proteins involved in polarity establishment and maintenance.**
(TXT)

Table S2 **The protein-protein interaction network data file downloaded from BioGRID database version 2.0.52.** This database file was changed slightly based on the following information: (1) For all the interactions happened in budding yeast polarity area, ACT1 is divided into two groups, according to its localization and funciton: **ACT1C** (actin cables), localized at the actin cable; and **ACT1P** (Actin patches), localized to the actin patch, to interact with its interaction partners. (2) ARP2 and ARP3 are included in the same complex called **ARP**. (3) Homologous proteins ENT1 and ENT2 are included in the same complex called **ENT1,2.** (4) SEC3, SEC5, SEC6, SEC8, SEC10, SEC15, EXO70, EXO84 are included in the same complex called **EXO**. (5) All proteins names in this table are in upper case. This is to keep consistent with the protein names in Biogrid database.
(TXT)

Table S3 **The 302 physical interactions among the 111 polarity proteins given in Table S1.**
(SIF)

Table S4 **The R codes to analze the two prostate cancer data sets GSE6919 and GSE32269 downloaded from GEO database.**
(DOCX)

Table S5 **All data sets used to find the key genes involved the metastasis prostate cancer.**
(DOCX)

Table S6 **The 3998 differentially expressed genes identified after the comparison between metastasis prostate cancer and normal tissue.**
(TXT)

Table S7 **List of 813 proteins in mouse for modulization and role determination.**
(TXT)

File S1 **User Guide: how to use the online and off-line version of ModuleRole.**
(DOCX)
