# Supplementary figures and images for: ModuleRole: A Tool for Modulization, Role Determination and Visualization in Protein-Protein Interaction Networks
Source: PLoS One. 2014 May 1;9(5):e94608. doi: 10.1371/journal.pone.0094608 (PMC4006751; doi:10.1371/journal.pone.0094608)

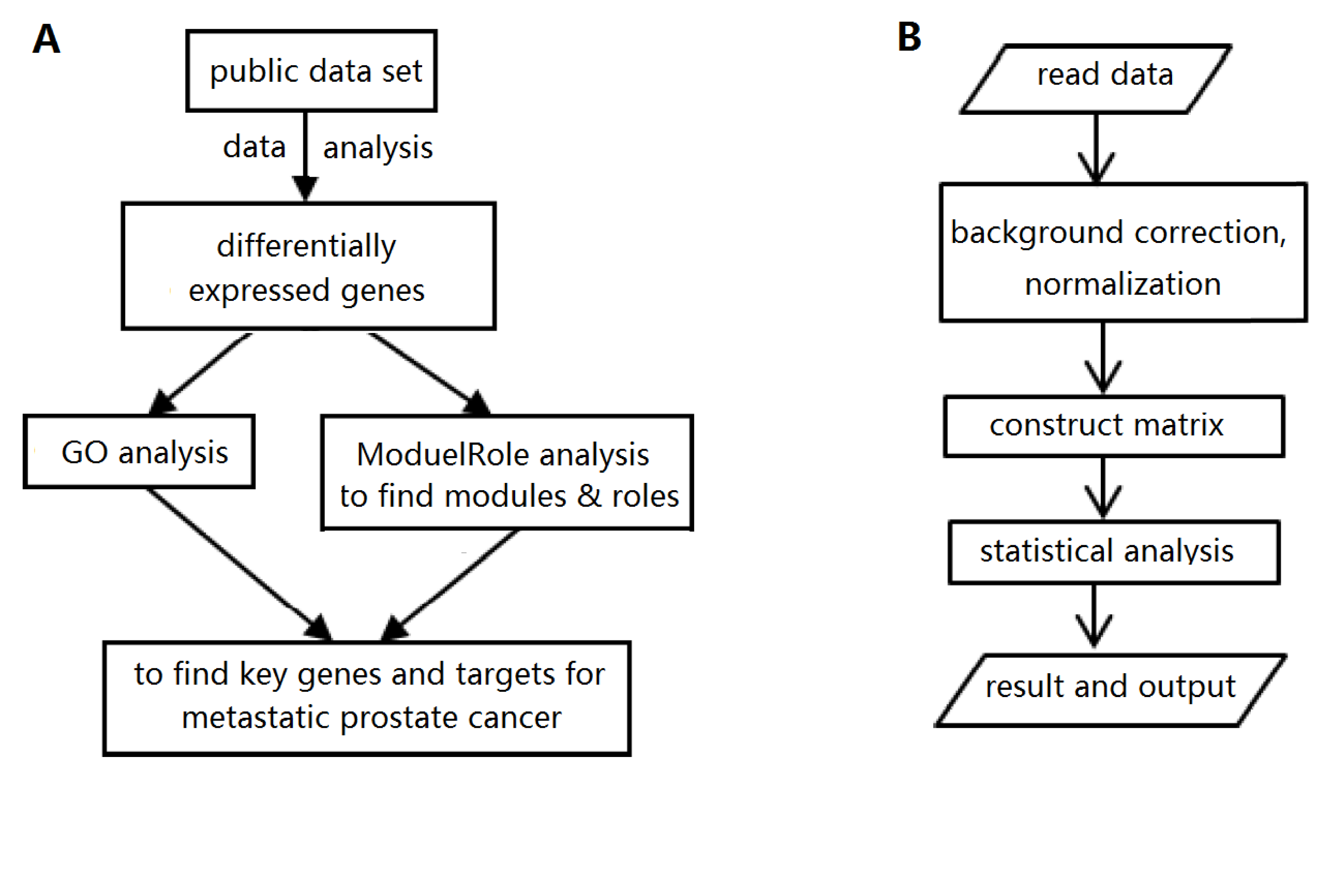

Supplement: Figure S1 — The pipline for the analysis of metastatic prostate cancer data. A. The workflow to find key genes for metastatic prostate cancer. B. The workflow to identify differentially expressed genes in data set GSE6919 and GSE32269. (TIF) [file pone.0094608.s001.tif]

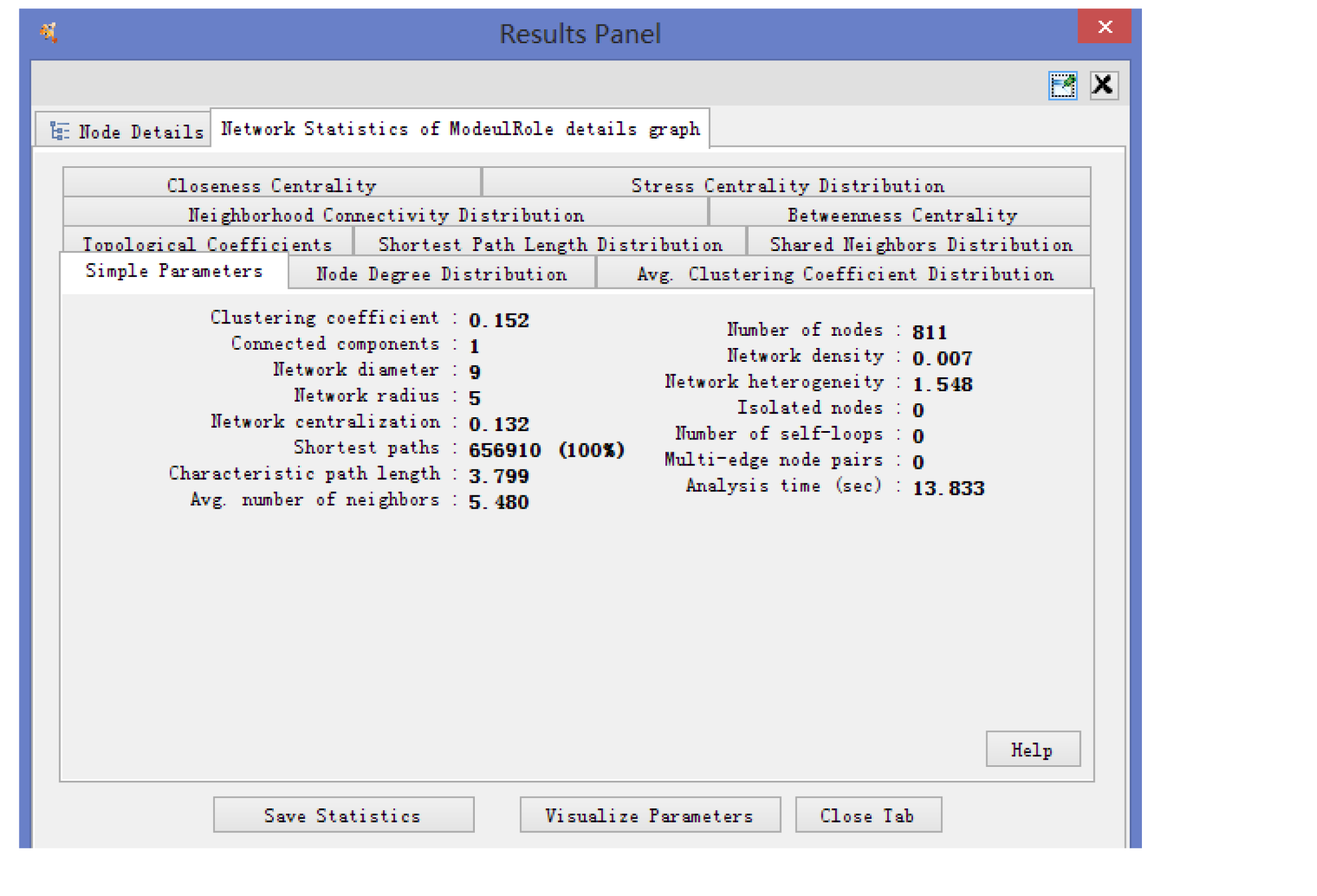

Supplement: Figure S2 — The plugin NetworkAnalyzer was used as an example to further analyze the xgmml file loded into Cytoscape. (TIF) [file pone.0094608.s002.tif]

**Table S4**. The R codes to find differentially expressed genes in data set GSE6919 and GSE32269:


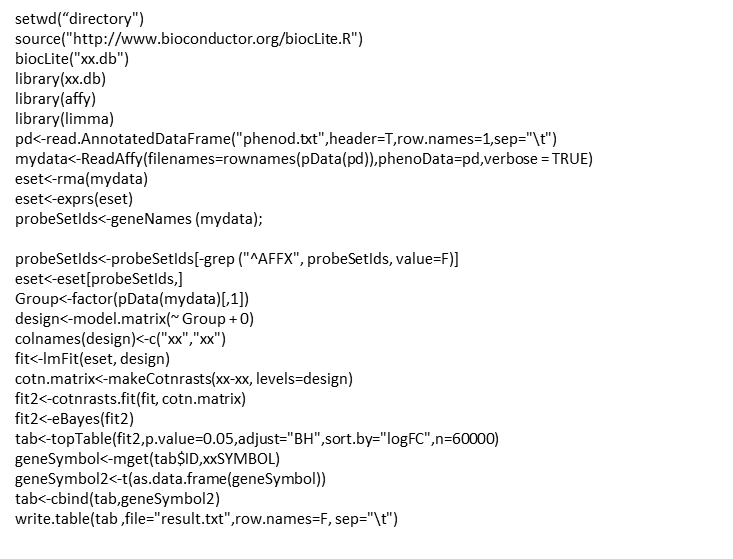

Supplement: Table S4 — The R codes to analze the two prostate cancer data sets GSE6919 and GSE32269 downloaded from GEO database. (DOCX) [file pone.0094608.s006.docx]
