# Supplementary material for: ModuleRole: A Tool for Modulization, Role Determination and Visualization in Protein-Protein Interaction Networks
Source: PLoS One. 2014 May 1;9(5):e94608. doi: 10.1371/journal.pone.0094608 (PMC4006751; doi:10.1371/journal.pone.0094608)
Supplement: Table S5 — All data sets used to find the key genes involved the metastasis prostate cancer. (DOCX) [file pone.0094608.s007.docx]

**Table S5.** The details of three datasets to define the genes key to metastatic process

| Data set | Data source | Sample details | Sample source | samples |
| --- | --- | --- | --- | --- |
| GSE6919 | NCBI | Metastatic Prostate Tumor | tissue | 25 |
|  |  | Primary Prostate Tumor | tissue | 65 |
|  |  | Normal prostate tissue | tissue | 18 |
| GSE32269 | NCBI | Metastatic Prostate Tumor | tissue | 29 |
|  |  | Primary Prostate Tumor | tissue | 22 |
|  |  | normal bone marrow sample | tissue | 4 |
