# Supplementary material for: ModuleRole: A Tool for Modulization, Role Determination and Visualization in Protein-Protein Interaction Networks
Source: PLoS One. 2014 May 1;9(5):e94608. doi: 10.1371/journal.pone.0094608 (PMC4006751; doi:10.1371/journal.pone.0094608)
Supplement: File S1 — User Guide: how to use the online and off-line version of ModuleRole. (DOCX) [file pone.0094608.s010.docx]

**User Guide**

1. Introduction
   1. The interface of ModuleRole
   2. Online and offline version of ModuleRole
2. How to use the online ModuleRole
   1. Input
   2. Output
   3. Visualization
3. How to install ModuleRole
4. Example: Demo data
5. Q&A about several methodological points
6. **Introduction**

In order to gain insight into the organization and structure of the resultant large complex networks formed by interacting molecules, using simulated annealing, a method based on the node connectivity, we developed ModuleRole, a user-friendly web server tool which finds modules in PPI network and defines the roles for every node, and produces files for visualization in Cytoscape and Pajek. For given proteins, it analyzes the PPI network from BioGRID database, finds and visualizes the modules these proteins form, and defines the role every node plays in this network, based on two topological parameters Participation Coefficient and Z-score. The description of the identified roles and their potential biological meaning can be found in Table 1 in the next page.

This is the first program which provides interactive and very friendly interface for biologists to find and visualize modules and at the same time, the roles of proteins in PPI network. It can be tested online at the website http://www.bioinfo.org/modulerole/index.php, which is free and open to all users with no login requirement.

Non-server application of this program is considered for high-throughput data with protein node number >=200 or user’s own interaction datasets. Users are able to bookmark the web link to the result page and access at a later time. As an interactive and highly customizable application, ModuleRole requires no expert knowledge in graph theory on the user side, thus a useful tool for biologist to analyze and visualize PPI networks in databases such as BioGRID. It can be used in both Linux and Windows system.

**Table 1**. The description of the identified roles and their potential biological meaning.

| **Role** | **Node type** | **Hub or non-hub** | **How is this node connected** | **Potential biological meaning** |
| --- | --- | --- | --- | --- |
| 1 | ultra-peripheral nodes | Non-hub | nodes with all their links within their module | Redundant with other proteins, or has a paralog. When deleted, the cell or species can survive well even without any phenotype changes. |
| 2 | peripheral nodes |  | nodes with most links within their module |  |
| 3 | non-hub connector nodes |  | nodes with many links to other modules | Protein interacts with proteins in two or several different pathways. |
| 4 | non-hub  kinless nodes |  | nodes with links homogeneously distributed among all modules | Protein involved in many pathways but does not play key role. This kind of node seldom appears. |
| 5 | provincial hubs | Hub | hub nodes with vast majority of links within their module | Essential protein plays key role in one specific pathway. |
| 6 | connector hubs |  | hubs with many links to most of the other modules | Essential protein plays key role in several pathways. |
| 7 | kinless hubs |  | hubs with links homogeneously distributed among all modules | Seldom appears. Protein involved in many pathways and play key role in several pathways. This kind of node seldom appears. |

**1.1 The interface of ModuleRole:**

The interface is from http://www.bioinfo.org/modulerole/index.php:


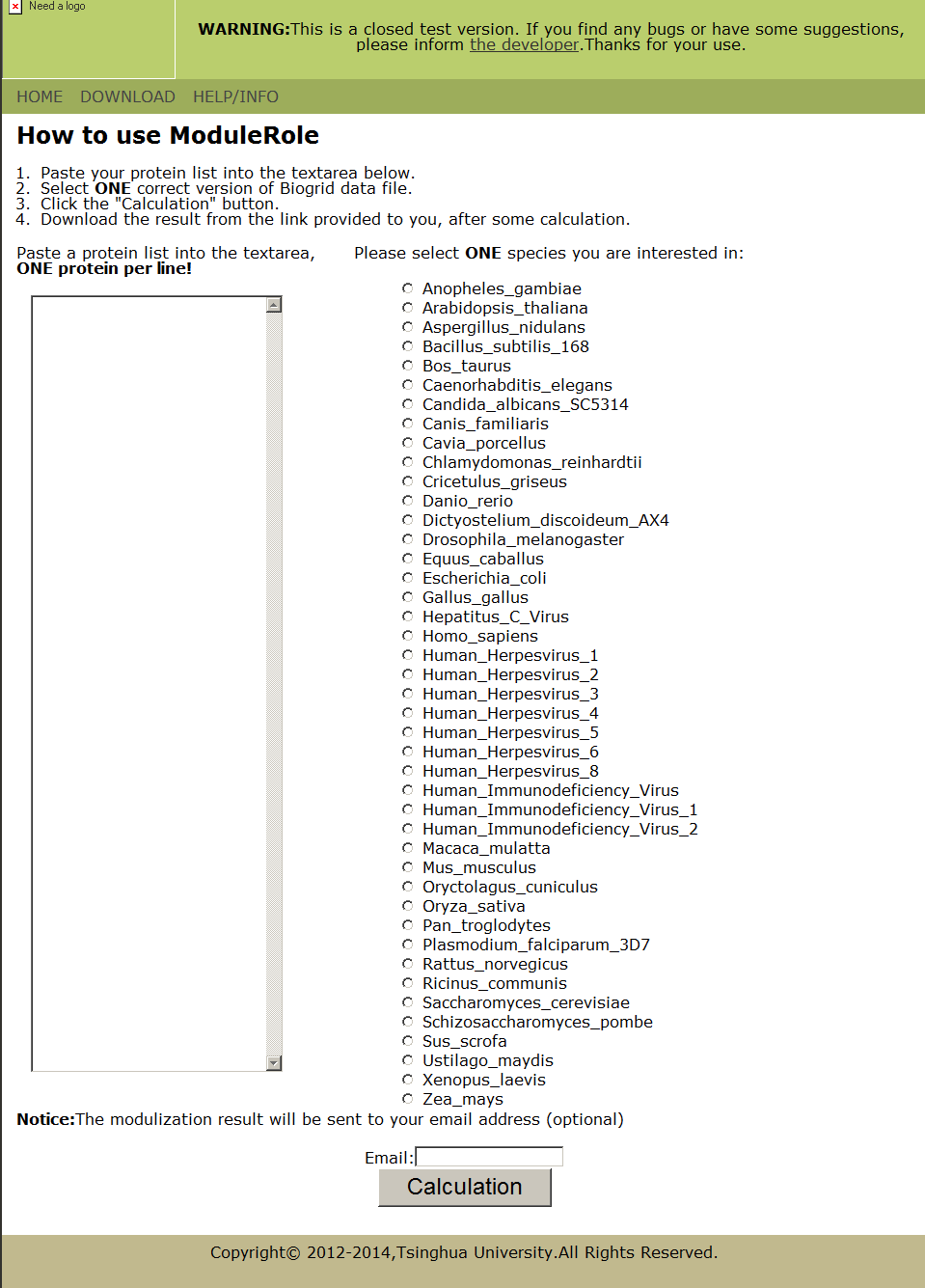


Figure 1. The interface of ModuleRole.

**1.2 The online and offline version of ModuleRole:**

The PPI network with nodes less than 200 can be analyzed by the online version. For the network more than 200 nodes, user needs to download the stand-alone version of ModuleRole and run this program offline.

1. **How to use the online ModuleRole**

**2.1 Input**

The program requires (1) a list of protein names defined by user and (2) a tab file downloaded directly from BioGRID which includes all species’ interaction data stored in BioGRID. Users can also simply select one species in the list on the right side of the interface (Figure. 1), which contains the latest version of BioGRID PPI data. For users who are interested in the data of older version, please download it directly from [www.biogrid.com](http://www.biogrid.com).

**2.2 Output**

The output has been designed to detect and visualize the details of interactions in every module, and the role assigned to every node. As ModuleRole is designed to analyze both physical PPI network and genetic PPI network, the output can be in three folders as shown in the following.


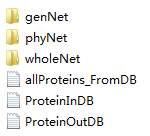


Folder **genNet** includes all result files for genetic PPI network, **phyNet** includes all result files for physic PPI network, while **wholeNet** includes all result files for the mixed networks of both genetic and physical interactions.

**allProteins_FromDB.txt** lists all proteins involved in either physical or genetic or both interactions in the species in BioGRID database used by user.

**ProteinOutDB.txt** lists the proteins given by user, but not found in the allProteins_FromDB.txt, i.e. not found in the PPI network in the version of BioGRID database used by user. These proteins might not exist in the given proteome, or just typos by user. This file is empty if all of the proteins in the list given by user can be found in allProteins_FromDB.txt.

**ProteinInDB.txt** lists the proteins given by user, which can be found in the allProteins_FromDB.txt.

**Output directory structure** of all results is shown below (We take **physical** PPI network as an example to demonstrate the result of Modulization and role determination). All results are in either phyNet\simulatedAnnealing, genNet\simulatedAnnealing, or wholeNet\simulatedAnnealing:

**
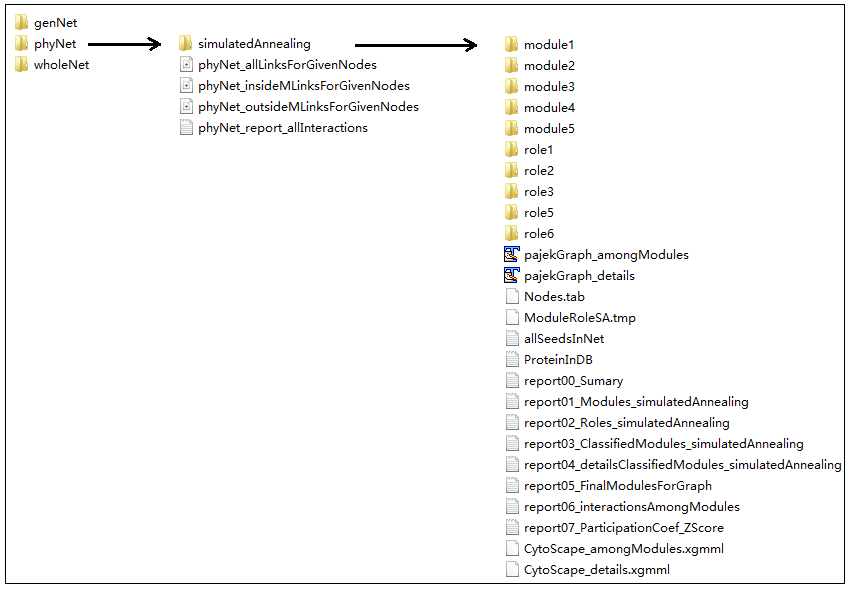
**

**Figure 2. The directory structure of ModuleRole output.**

In folder phyNet\simulatedAnnealing, the output can be divided into five parts, as shown in Figure 3. We take budding yeast cell polarity PPI network as an example. This network produces 5 modules and 5 kinds of roles.


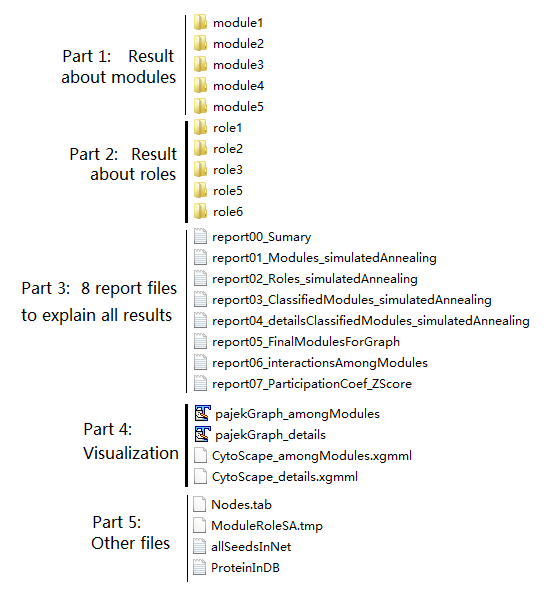


Figure 3. The five parts of result files in ModuleRole output directory.

1. **Part 1: result about modules：**

Part 1 includes all subfolders about modulization. Every module is assigned a number and a subfolder, in this case, module 1 to 5. In every subfolder, two files were produced. the one named as “polarityModule*.txt” (* is the module number) is to show all details in this module, including:

**Nodes Number in this module**;

**Total Link Number in this module**;

**Within-Module link Number in this module**, which indicates how many links INSIDE of given module;

**The Number of links from this module to other modules**, which indicates how many links OUTSIDE of given module;

And **the list of proteins included in this module**.

The other file named as “seedsPList_module*.txt” is the list of proteins included in this module, but in the form of a column.

The output of the list of proteins in every module was made in two forms: one is in a row (as seen in the file “polarityModule*.txt”), while the other in a column (as seen in the file “seedsPList_module*.txt”). These two forms help users copy and paste the list of proteins in a very convenient way.

1. **Part 2: result about roles：**

In this part, all proteins which have the same role belong to one subfolder. For the case of yeast polarity PPI network, there are 5 different kinds of roles, thus 5 subfolders. In every subfolder, there are two files: “polarityRole_*.txt” and “seedsPList_role*.txt” (* is the role number).

In the file “polarityRole_*.txt”, there are three parts: Role number, the number of nodes with that role, and the list of proteins which have that role.

In the file “seedsPList_role*.txt”, there is a list of proteins with given role, but in the form of column.

1. **Part 3: Eight report text files** to show the details of modulization and role determination:
   1. **Report00_Summary.txt** summarizes the calculation of the data set (in this case the yeast polarity PPI network). The summarization includes: what is modularity value, how many proteins were used for the modulization and role determination, how many interactions are there in the PPI network given by user, etc.
   2. The following two files show the details about the modules and roles defined by the program:

**report01_Modules_simulatedAnnealing** shows the proteins in every module defined by ModuleRole.

**report02_Roles_simulatedAnnealing** shows every group of proteins which have the same role defined by the algorithm.

- 1. The following two files show the details about the interaction number every protein has in every module:

**report03_ClassifiedModules_simulatedAnnealing** shows the interaction numbers every protein has in every module.

**report04_detailsClassifiedModules_simulatedAnnealing** shows that for every protein, how the interaction number in report03_ClassifiedModules_simulatedAnnealing.txt are defined.

- 1. Report 5 and 6 shows the details about the interaction numbers every module has with other modules:

**report05_FinalModulesForGraph** shows the interaction numbers among different modules. This file can be treated as the **table version** of the NET file pajekGraph_amongModules.net which can be used for visualization in Pajek.

**report06_interactionsAmongModules** shows that for every module, how the interaction numbers in report05_FinalModulesForGraph are defined.

- 1. ModuleRole enables users to examine, for instance, structural parameters such as Participation Coefficient and Z-score in a network. This is shown in the report file **report07_ParticipationCoef_ZScore.txt**.

1. **Part 4: Visualization**

**Two xgmml files** are to visualize the modules and roles in Cytoscape:

- 1. **CytoScape_details.xgmml** is to visualize the proteins in every module and the role of every protein defined by ModuleRole.
  2. **CytoScape_amongModules.xgmml** is the coarse graining graph, that is, the simplified graph, of the whole network, to help user gain insight about the contribution of the links between every two modules to the biological process user is interested in.

**Two NET files** are to visualize the modules and roles in Pajek:

- 1. **pajekGraph_details.net** is to visualize the proteins in every module and the role of every protein defined by ModuleRole.
  2. **pajekGraph_amongModules.net** is the coarse graining graph, that is, the simplified graph, of the whole network, to help user gain insight about the contribution of the links between every two modules to the biological process user is interested in.

5. **Part 5: Other files**, which are useful for developers, thus can be overlooked by users.

**2.3** **Visualization**

For the visualization in Cytoscape, user needs to load xgmml files into Cytoscape. At first user needs to install Cytoscape from <http://www.cytoscape.org/download.html>. After Cytoscape is running, press the third button on Cytoscape panel (the button marked in red, see the following), to choose one of the two xgmml files produced by ModuleRole in the dialog window:


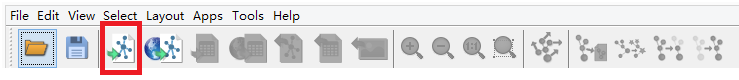


After loading the file, user needs to select what layout she/he prefers. One choice is “Layout/Group Attributes Layout/Module” and the visualization of mouse PPI network will be the following (Figure. 4):


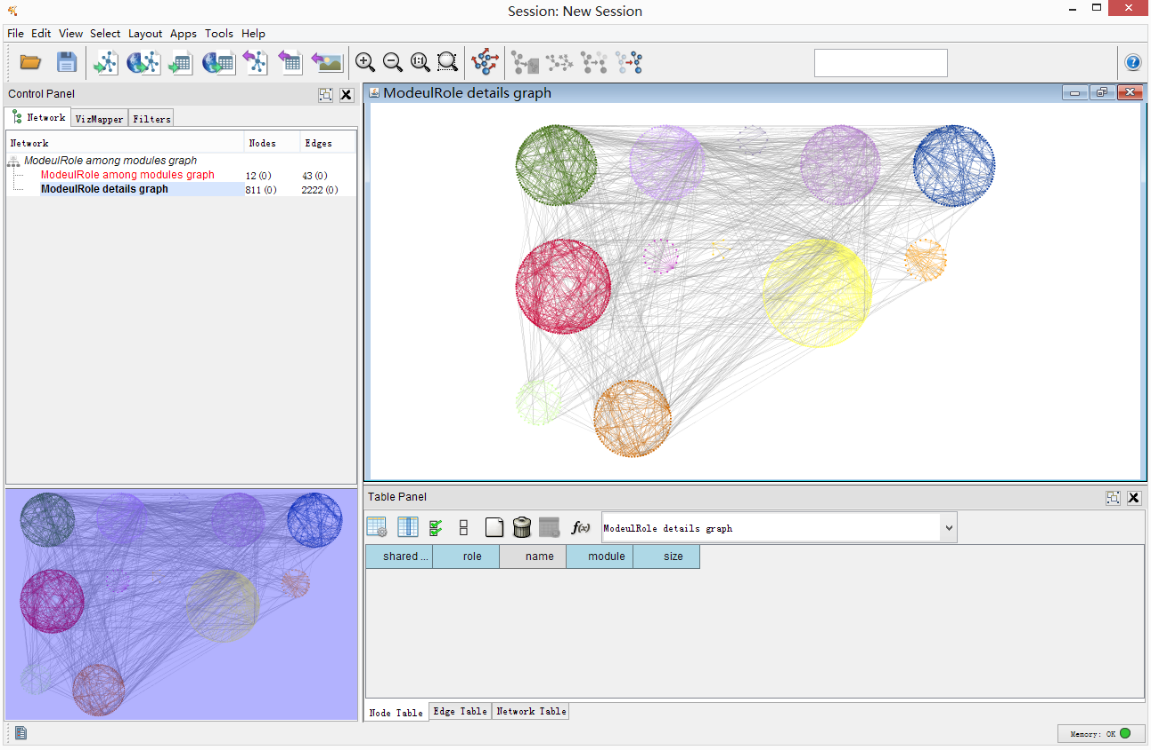


Figure 4. The visualization of modules and roles of mouse PPI network with the “Group Attributes Layout/Module” layout.

User can also use other powerful plugins in Cytoscape to do quantitative analysis of biological networks, after the input of the xgmml file. One example is plugin NetworkAnalyzer, which is used for the standard and advanced analysis of network topologies. User can choose Tools/NetworkAnalyzer/Network Analysis/Analyze Network, as following,


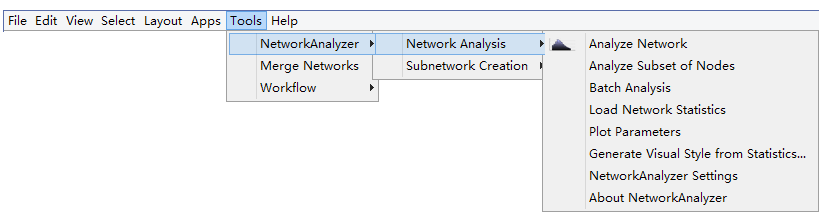


to get the quantitative result about the mouse physical PPI network (see Figure. 5):


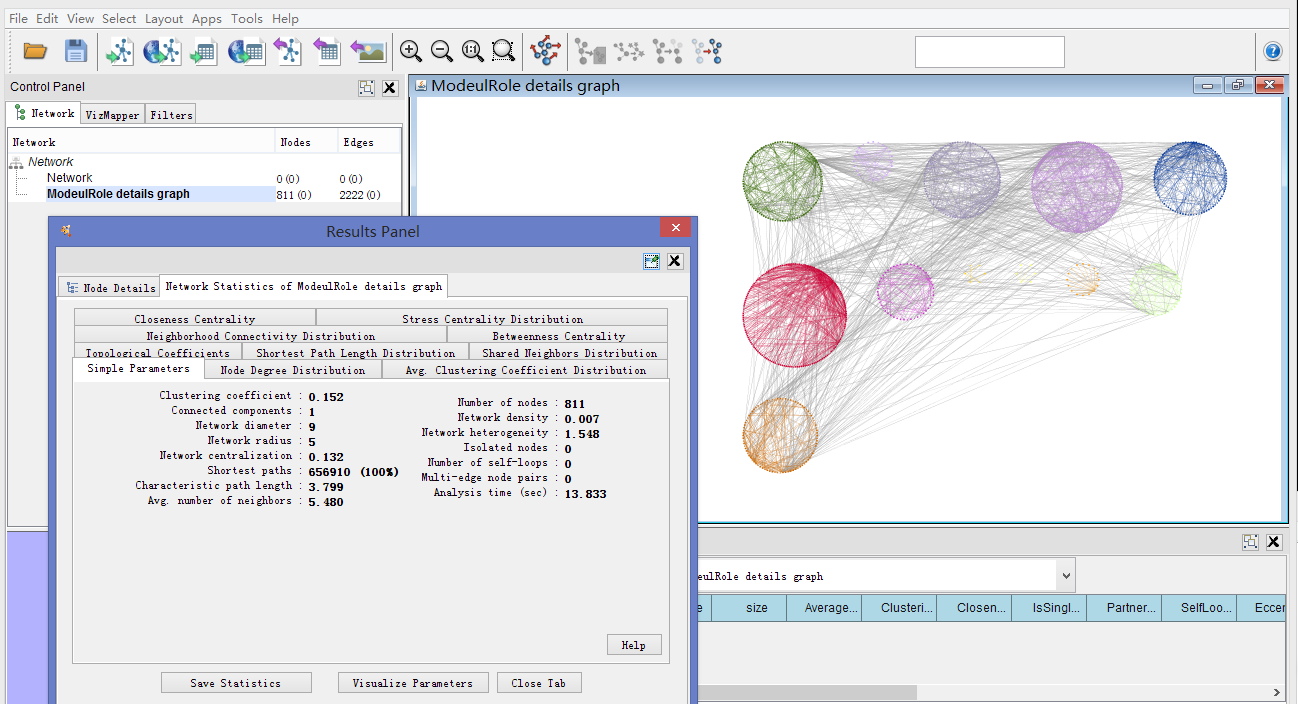


Figure 5. The quantification of mouse PPI network, with xgmml files produced by ModuleRole as input for Cytoscape.

For the visualization in Pajek, users need to download the visualization software Pajek from <http://vlado.fmf.uni-lj.si/pub/networks/pajek/>. The input for Pajek are the two *.NET files produced by ModuleRole. After Pajek is running, under the red label “Networks”
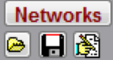
, click the first button
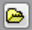
 to select **pajekGraph_details.net.** In menu **Draw**, select **draw** (user can also use Ctrl+G instead) to visualize the network, see the following:


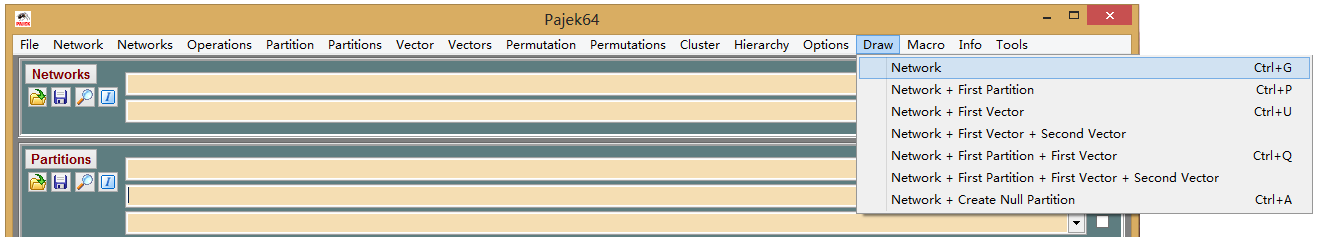


1. **How to install the offline version of ModuleRole**

Non-server application of ModuleRole is considered for high-throughput data --- user can download ModuleRole and run it offline for large data set with protein node number >=200 -- or user’s own interaction datasets. For this purpose, ModuleRole can be downloaded from http://www.bioinfo.org/modulerole/download.php. This is a command line version which is designed to run on local server (Linux or Windows system). Once it is downloaded, user can put it in the folder user prefers and run it without further installation, except that java environment is required (java 1.7 is preferred).

For Java Installation, please visit Java official website <http://www.oracle.com/technetwork/java/javase/downloads/index.html> to download the latest version of JDK (Java Development Kit) and/or JRE (Java Runtime Environment). In Windows Operating System, users need to set two Environment Variables: path and CLASSPATH, to let Windows know where java.exe is and where ModuleRole classes are.

For the installation of ModuleRole under Linux Operating System, GSL (GNU Scientific Library) is required.

After Java installation, users will use the following command to run ModuleRole on command line:

**Usage:** *java –jar ModuleRole.jar Absolute_Path_of_BioGRIDdatafile Absolute_Path_of_ProteinList Absolute_Path_of_OutputResultDirectory*

In this command, three files are used:

1. *Absolute_Path_of_BioGRIDdatafile*: is the BioGRID database file plus its absolute pathway. For example, the BioGRID database file can be: [BIOGRID-ORGANISM-3.2.95.tab.zip](http://thebiogrid.org/downloads/archives/Release%20Archive/BIOGRID-3.2.95/BIOGRID-ORGANISM-3.2.95.tab.zip) which can be downloaded from [http://theBioGRID.org/download.php](http://thebiogrid.org/download.php) and then unzipped the zip file to any path user prefers.
2. *Absolute_Path_of_ProteinList*: is the ProteinList file plus its absolute pathway. The ProteinList file is a txt file which contains the proteins user is interested in. The format of this file is one column of protein list, for example:

ARK1

AXL2

BAG7

BOI1

BUD14

BUD2

…

1. *OutputResultDirectory* is the directory which user wants to save the output results of ModuleRole.
2. An example for this command is given here:

*java –jar /my/ModuleRole.jar /my/DB/BIOGRID-ORGANISM-Homo_sapiens-3.2.95.tab.txt /my/proteinlist.txt /my/output_result_directory*

1. **Three ways to launch the command prompt in Windows 8:**
2. For users who don’t know how to launch the command prompt in Windows Operating System, there are three ways to do that:
   **Option 1**: To open the RUN dialog, press the Windows key and then press "R". This will open the RUN dialog for user. To open the command prompt from here, type "cmd" and press ENTER.

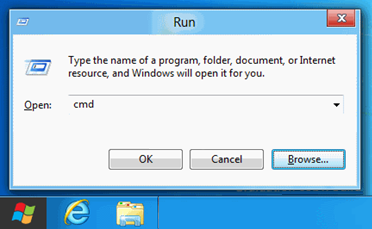


**Option 2:** When user is in the Metro UI (See the picture below), start typing "cmd". It will automatically go in to search mode and will search for what user typed (cmd).
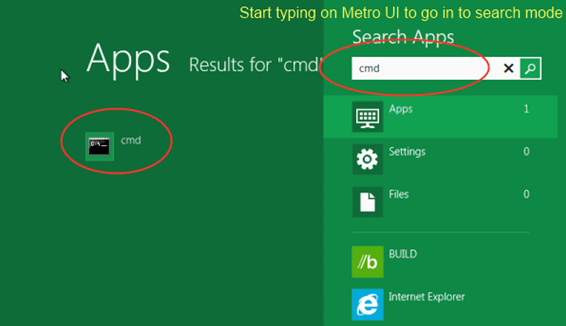


**Option 3**: User can open the command prompt by starting the cmd.exe in the windows system32 folder. (1) Open Windows Explorer. (2) Navigate to the folder C:\Windows\System32. (3) Look for the application CMD.EXE and double click on it.
It will open the command prompt, which looks exactly same as mentioned above. User can double click on cmd.exe and choose "Pin to Taskbar" which will add a shortcut to the taskbar for easy access to the command prompt in future.

1. **Example: Demo data**

Here we take the polarity PPI network in budding yeast as an example. The following are 99 proteins which form a PPI network important for cell polarity establishment and maintenance:

ARK1

AXL2

BAG7

BOI1

BUD14

BUD2

BUD5

BUD8

BZZ1

CAP2

CDC55

CHC1

GIC1

GYL1

GYP5

KEL2

LTE1

MKK1

MSB2

MSO1

PAM1

PXL1

RGD2

RHO2

RHO4

RHO5

ROM2

RVS161

SAC6

SCP1

SEC1

SEC9

SMY1

SNC1

SNC2

SPH1

SSO1

SSO2

VRP1

YAP1802

YOR304C-A

ABP1

ACT1C

ACT1P

ARP

BBC1

BEM1

BEM3

BNI1

BOI2

BUD6

CAP1

CDC24

CLA4

CLC1

CLN2

CMD1

COF1

END3

ENT1,2

GIC2

KEL1

LRG1

MSB1

MSB3

MSB4

MYO2

MYO3

MYO4

MYO5

PAN1

PEA2

PKC1

PRK1

RGA1

RGA2

RGD1

RHO1

RHO3

RSR1

RVS167

SEC4

SHE3

SLA1

SLA2

SLG1

SPA2

SRO7

SRO77

TOS2

YFR016C

YMR124W

BEM2

CBK1

EXO

SEC2

STE20

LAS17

CDC42

At first we paste these 99 proteins into the text area on the webpage (see Figure. 1). Next we select one species “Saccharomyces_cerevisiae” and press the button “calculation”. After some seconds, a new webpage appears to show the link of result.zip file. Download and open this zip file, we will find:


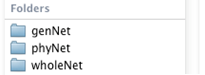


Folder “genNet” includes the module/role result of the GENETIC interaction network of these 99 proteins. Folder “phyNet” shows the modules/role result of the PHYSICAL interaction network of these 99 proteins. Folder “wholeNet” shows the modules/role result of the WHOLE network, which includes both GENETIC and PHYSICAL interactions of these 99 proteins.

Here we take physical PPI network result (in folder “phyNet”) as an example. Double click “phyNet”, we will see:


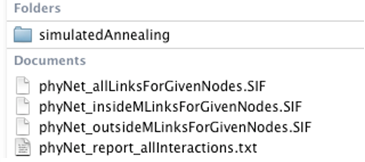


**phyNet_allLinksForGivenNodes.SIF** shows all physical interactions between the given 99 proteins and budding yeast proteome,

**phyNet_insideMLinksForGivenNodes.SIF** shows all physical interactions among the given 99 proteins,

**phyNet_outsideMLinksForGivenNodes.SIF** shows all physical interactions between the given 99 proteins and all OTHER proteins in budding yeast proteome.

The results are in folder “simulatedAnnealing”:


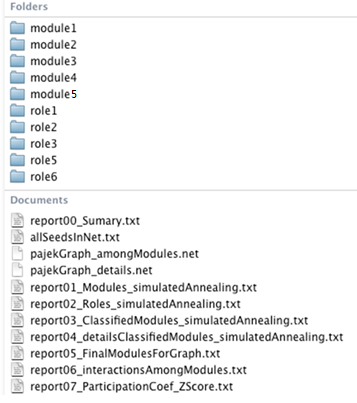


All 99 proteins are divided into five modules, which are named module 1, 2, 3, 4, 5 respectively. The **two** ***.NET files, two *.xgmml files and the eight report*.txt file**s are the result we need. All 99 proteins are assigned five roles, which are role 1, 2, 3, 5, 6 respectively.

For the visualization of the modulized network, user can use either Cytoscape or Pajek. For the case of Pajek, user needs to load **pajekGraph_details.net** into Pajek:

Open Pajek, under the red label “Networks“
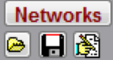
, click the first button
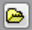
 to select **pajekGraph_details.net.** In menu **Draw**, select **draw** (user can also use Ctrl+G instead) to visualize the network as following:

**
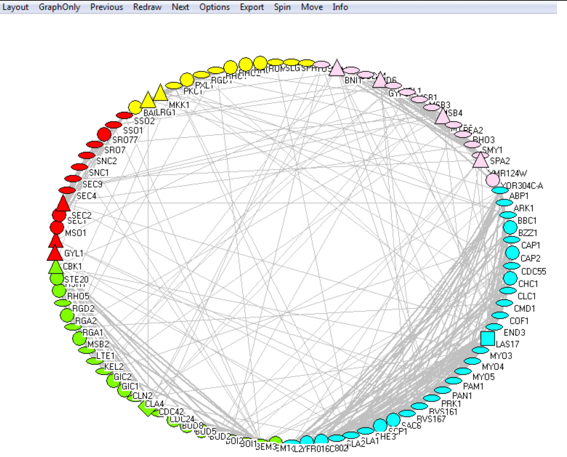
**

Every color indicates one module. Use can change the layout of the modules and nodes manually or automatically.

User can also load **pajekGraph_amongModules.net** into Pajek and get the following visualization:


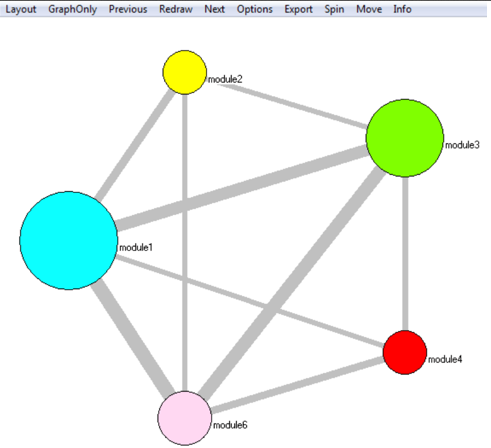


Here the size of the nodes indicates the protein number in the module, the width of every line indicates the interaction number between two modules at the line end, and the color corresponds to the node color in the protein network for pajekGraph_details.net.

1. **Q&A about several methodological points**
2. Could every user supplied protein list be used to obtain meaningful results?

User will get very meaningful result if the input of ModuleRole, i.e., user supplied protein list and BioGRID database file, satisfies the following condition: The BioGRID database file contains ENOUGH knowledge about PPI network reconstructed from user supplied protein list. What we need to point out is, for users’ own interaction datasets, only the offline version of ModuleRole can be used.

1. Are there some minimal/maximal length, or connectivity constraints for the protein list supplied by user?

There is no limit for the length of the user supplied protein list. The number of proteins input by user can be from zero to the number of all proteins in the BioGRID database file used by user.

1. Why and when the cluster structure obtained from the program is expected to be informative?

As a biologist, user knows exactly whether the modulization and role determination defined by ModuleRole is meaningful or not, and what kind of hint the result can provide, at what level. This requires that user has good knowledge of biology and of the field she/he is studying.

1. How does the detected community structure correlate with the global network structure?

Community structure can be obtained through the division of network nodes into groups within which the network connections are dense, but between which are sparser. The ability to find and analyze such community structure can provide invaluable help in understanding and visualizing the global network structure. However, global network structure is not the focus of the work finished by ModuleRole.

1. Why does the modulization and role determination produced by ModuleRole slightly change when I run ModuleRole sometimes?

To implement simulated annealing, which was used by ModuleRole for modulization, it is usually necessary to generate huge amounts of random numbers, which keeps changing every time. Thus the modulization result changes slightly when user runs the program several times. For example, protein A is in module 1 this time, but next time, it moves to module 2, and the third time, it moves back to module 1. This is normal for the proteins at the “border” of two modules (See the Supplementary Figure S2 of the paper Gao et al 2011(PNAS, 108: 7647–7652) for more details). But for the proteins not at the “border”, the result will keep the same. Therefore, please don’t get confused about the slight change of the results. How to identify robust modules was discussed and illustrated in Supplementary Figure S2 of the paper Gao et al 2011(PNAS, 108: 7647–7652).

For the different version of BioGRID database as input, the modulization and role determination result might also be slightly different.
